# Evaluation of low-dose aspirin for primary prevention of ischemic stroke among patients with diabetes: a retrospective cohort study

**DOI:** 10.1186/s13098-015-0002-y

**Published:** 2015-02-15

**Authors:** Ye-Jee Kim, Nam-Kyong Choi, Mi-Sook Kim, Joongyub Lee, Yoosoo Chang, Jong-Mi Seong, Sun-Young Jung, Ju-Young Shin, Ji-Eun Park, Byung-Joo Park

**Affiliations:** Korea Institute of Drug Safety and Risk Management, Seoul, Republic of Korea; Medical Research Collaborating Center, Seoul National University College of Medicine/Seoul National University Hospital, Seoul, Republic of Korea; Institute of Environmental Medicine, Seoul National University Medical Research Center, , Seoul, Republic of Korea; Department of Preventive Medicine, College of Medicine, Seoul National University, Seoul, Republic of Korea; Center for Cohort Studies, Total Healthcare Screening Center, Kangbuk Samsung Hospital, Sungkyunkwan University College of Medicine, Seoul, Republic of Korea; National Evidence-based Healthcare Collaborating Agency, Seoul, Republic of Korea; Department of Health Care Management and Policy, School of Public Health, National University, Seoul, Republic of Korea

**Keywords:** Aspirin, Diabetes mellitus, Ischemic stroke, Health insurance claims database, Retrospective cohort study

## Abstract

**Background:**

Low-dose aspirin is recommended to reduce the risk of cardiovascular disease. However, the questions with regard to primary prevention have been raised among patients with diabetes. We evaluated low-dose aspirin use for preventing ischemic stroke in patients with diabetes using a national health insurance database.

**Methods:**

Using data from the Korean Health Insurance Review and Assessment Service database from January 1, 2005, through December 31, 2009, a population-based retrospective cohort study was conducted with incident patients with diabetes aged 40 to 99 years old with the initial use of low-dose aspirin during the index period from January 1, 2006 to December 31, 2007. We matched each low-dose aspirin user to one non-user using a propensity score. The Cox proportional hazards model was used to compare the risk of hospitalization for ischemic stroke in users and nonusers of low-dose aspirin until December 31, 2009.

**Results:**

Out of 261,065 incident patients with diabetes, 15,849 (6.2%) were low-dose aspirin users. Compared to non-users, the adjusted hazard ratio (95% confidence interval) of low-dose aspirin users for hospitalization due to ischemic stroke was 1.73 (95% CI; 1.41-2.12). In a sensitivity analysis of study subjects with more than 1 year follow-up periods, slightly higher adjusted hazard ratio (1.97, 95% CI; 1.51-2.62) was observed. In the subgroup analyses, there were no significant changes in the risk of hospitalization for ischemic stroke irrespective of gender, age, or comorbidity.

**Conclusions:**

In this study of patients with diabetes, the use of low-dose aspirin showed an increased risk of hospitalization for ischemic stroke. These results suggest that low-dose aspirin use for the primary prevention of ischemic stroke should be reconsidered in patients with diabetes.

## Background

Low-dose aspirin is recommended to prevent cardiovascular morbidity and mortality in high risk patients with myocardial infarction or ischemic stroke for the purpose of secondary prevention. It is also recommended to prevent cardiovascular events in the adult general population without a previous history of cardiovascular disease [1].

However, the Primary Prevention Project (PPP) trial raised doubt about the effectiveness of aspirin in diabetes patients [2]. Two subsequent randomized controlled trials that enrolled only patients with diabetes did not show a benefit of low-dose aspirin in primary prevention for cardiovascular disease, but did report an increased risk of gastrointestinal bleeding [3,4]. A meta-analysis by the Antithrombotic Trialists’ Collaboration provided subgroup analyses including patients with diabetes and found that the effects of low-dose aspirin on major vascular events were not statistically significant [5]. Furthermore, another meta-analysis including diabetic patients without cardiovascular disease found no significant reduction in the risk of major cardiovascular events with low-dose aspirin [6]. However, the previous studies were not sufficient to identify the effect of primary prevention due to their small sample size, no information on the use of statins, low level of compliance to aspirin therapy, switching aspirin use status during follow-up, or lack of precision of outcome measures [2-4,6]. Based on the limited evidence available, recent guidelines for managing diabetes mellitus narrow down the target of low-dose aspirin use for primary prevention to include only men aged over 50 years and women aged over 60 years, with an additional risk factor [7]. However, questions have been constantly raised about the use of low-dose aspirin on primary prevention of cardiovascular disease among patients with diabetes. Well-designed real world studies could be help to provide evidence determining the treatment effect with assessing the impact of age, gender or comorbidity. We attempted to evaluate the risk of ischemic stroke preferentially, because incidence rate for ischemic stroke was greater than any other cardiovascular diseases among patients with diabetes [8]. The objectives of this study were to evaluate the preventive effects of low-dose aspirin for ischemic stroke among the incident patients with diabetes compared with non-users.

## Methods

### Ethical approval

This study was approved by the Institutional Review Board of the National Evidence-based Collaboration Agency (NECA IRB09-011-2).

### Study design and data source

A retrospective cohort study design was used to evaluate low-dose aspirin use for preventing ischemic stroke in patients with diabetes.

This study was conducted using the Health Insurance Review and Assessment Service (HIRA) database. The HIRA database includes all information on approximately 50 million people, whole Korean population covered by the National Health Insurance (NHI) program since 2000. Every resident in South Korea is provided with a unique civil registration number. After with the NHI program provides coverage for all residents with the form of compulsory social insurance, which ensured the complete follow-up of study participants. We obtained claims data for patients that had been submitted by healthcare providers between January 1, 2005 and December 31, 2009 with anonymized identifiers given by HIRA to protect privacy, according to the Act on the Protection of Personal Information Maintained by Public Agencies. The database contains information on demographic records, diagnosis, procedures performed, and prescriptions. Demographic information included age, gender, and national health insurance type. The health insurance type was composed of two parts, health insurance based on employment or residential area and a separate program for the lower-income Medicaid group, which covered 3-5% of the whole population [9]. All diagnoses were coded using the Korean Classification of Disease, fifth edition (KCD-5) modification of the International Classification of Disease and Related Health Problems, 10th revision (ICD-10). Procedures were entered into the database based on the HIRA coding system. The prescription data included the brand name and generic name of the drug according to the HIRA drug formulary code, prescription date, and duration.

### Study population and exposure measure

**Figure 1:**
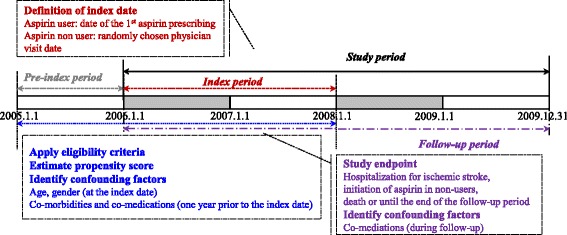
**Schematic description of the study period.**

The study cohort consisted of incident diabetes patients during the index period (January 1, 2006 to December 31, 2007), at 40 to 99 years of age at cohort entry (Figure 1). A patient with diabetes was defined as a patient diagnosed with diabetes (ICD-10, E10-14) and who had received a prescription for anti-diabetic medication(s) (oral hypoglycemic agents or insulin) in the same claim during the index period from January 1, 2006 to December 31, 2007. Because diabetes itself was regarded as a major risk factor for cardiovascular disease, we included incident patients with diabetes. To identify incident patients with diabetes, study participants were excluded if they had any claims of diabetes during the one year (January 1, 2005 to December 31, 2005) preceding the start of the index period. The study population was classified as low-dose aspirin (75–162 mg/day) users or non-users. The use of low dose aspirin was identified from the claims during the index period. The index date was defined as the date of low-dose aspirin initiation in low-dose aspirin users, and randomly chosen date among physician visit dates during the index period in non-users [10].

Patients were excluded from the study i) if they had a prescription of aspirin as an analgesic agent during the study period (January 1, 2005 to December 31, 2009), or ii) received low-dose aspirin before the index period, or iii) had already recorded ischemic heart disease (I20-25) or ischemic stroke (I63) or had been prescribed anti-thrombotic drugs (abcximab, argatroban, bemiparin, beraprost, cilostazol, clopidogrel, dalteparin, dipyridamole, enoxaparin, fondaparinux, heparin, iloprost, indobufen, lepirudin, limaprost, mesoglycan, nadroparin, ozagrel, reviparin, parnaparin, RH-tissue type plasminogen activator, sarpogrelate, streptokinase, sulodexide, sulfomucopolysaccharide, tirofiban, treprostinil, tenecteplase, ticlopidine, triflusal, urokinase, or warfarin; all the anti-thrombotic drugs covered by the HIRA during the study period) before the index period.

### Study outcome

The study outcomes of interest were defined as the principal and subsidiary diagnosis of hospitalization for ischemic stroke (I63).

### Follow-up

A patient was considered to be continuously exposed to low-dose aspirin if he or she filled a prescription within the end date of the previous prescription plus 1.5 times the prescription days’ supply [11]. Of the non-users, survival time was censored for those who initiated low-dose aspirin during the follow-up period (January 1, 2006 to December 31, 2009). All patients were followed until they experienced an ischemic stroke, initiation of aspirin in non-users, death, or until the end of the follow-up period (December 31, 2009), whichever came first. Date of death was identified by the ICD-10 codes (R96, R98, R99, and I46.1) that indicate patient death in the claims database.

### Statistical analyses

Baseline characteristics were presented as numbers with percentages for categorical variables and as means with standard deviations for continuous variables. We analyzed the patient characteristics including gender, age, insurance type, diabetes-related factors, co-morbidities, and use of medications in the year before the index date. The insurance type was classified as health insurance, Medicaid, or switching between the two groups. Diabetes-related factors included the type of diabetes and whether oral hypoglycemic agents or insulin was being used to control the diabetes. We defined type 1 diabetes-only patients as those who were recorded as having the ICD-10 code E10, or else classified as type 2 diabetes patients. Comorbidities were identified by ICD-10 codes for each patient, including essential hypertension (I10), and dyslipidemia (E78). Potential confounding factors were the use of statins, angiotensin converting enzyme inhibitors (ACEI), angiotensin receptor blockers (ARB), calcium channel blockers (CCB), beta blockers (BB), thiazide diuretics, and non-steroidal anti-inflammatory drugs (NSAIDs).

To reduce the effect of confounding by indication, we matched each low-dose aspirin user to one non-user on the basis of the propensity score, which was quantified as the likelihood of receiving low-dose aspirin in the year before the index date by multivariate logistic regression analysis [12]. In the model, the use of low-dose aspirin was used as a dependent variable, and all measured baseline patient characteristics as listed above were included in the analysis. We also tested the following clinically plausible interaction terms: age and co-morbidities, age and use of medications, as well as co-morbidities and use of medications. After calculating the predicted probabilities, we matched each low-dose aspirin user to one non-user using the Greedy 5-to-1 digit-matching algorithm [13]. Balances in the distribution of baseline covariates were estimated by the standardized difference between the two groups, before and after matching.

We performed a Cox proportional hazard analysis to evaluate the effects of low-dose aspirin on the incidence of ischemic stroke after adjusting for use of statins, ACEI, ARB, CCB, BB, thiazide diuretics, and NSAIDs during follow-up. The insurance type and anti-diabetic medications were adjusted since the unbalanced distribution was observed after propensity score matching. Hazard ratios and their 95% confidence intervals were calculated. The proportional hazard assumption was checked by examining the log-log plots of the hazard functions for each group. Subgroup analyses were conducted to explore the impact of each gender (male, female), age group (40–69, 70–99), type of diabetes (type 1 only, type2 and others), and presence of essential hypertension, dyslipidemia. To deal with confounding by indication, we also examined risk of hospitalization for ischemic stroke was associated with use of low-dose aspirin among study subjects with more than 1 year follow-up periods.

All statistical analysis was performed using SAS software (version 9.1, SAS Institute, Inc., Cary, NC, USA).

## Results and discussion

**Figure 2:**
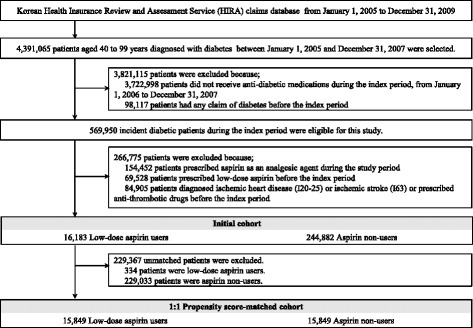
**Selection of the study participants from the Health Insurance Review and Sssessment Service database.**

From the HIRA database, 4,391,065 patients aged 40 to 99 years were diagnosed with diabetes between January 1, 2005 and December 31, 2009. Of these, we identified 569,950 incident patients with diabetes. After applying exclusion criteria, 261,065 patients were included in the initial cohort (6.2%) of whom were low-dose aspirin users and 93.8% of whom were aspirin non-users) and after matching by propensity score, 15,849 patients remained in each group (Figure 2).

The baseline characteristics of the study population before and after matching are presented in Table 1. Before propensity score matching, the users and the non-users widely differed on a number of baseline characteristics. Among the initial cohort, the low-dose aspirin users were more likely to be older, to have essential hypertension (48.9% vs. 23.1%) and dyslipidemia (26.3% vs. 11.3%), and to use of medication: statins (26.5% vs. 11.7%), ACEI (13.8% vs. 4.9%), ARB (26.7% vs. 10.0%), CCB (39.0% vs. 19.3%), BB (16.7% vs. 10.8%), and thiazide diuretics (27.7% vs. 12.6%). However, propensity score matched groups were balanced for gender, age group, type of diabetes, diagnosis of essential hypertension and dyslipidemia, and use of medications considered. Standardized differences in patient characteristics, which were below 0.1 across the groups, demonstrate substantial improvement in the balance of covariates [14].

**Table 1:** **Baseline characteristics of study cohort by use of low**-**dose aspirin before and after propensity score matching**

**Characteristic** ^*^		**Initial cohort**					**Propensity score matched cohort**				
		**Aspirin non**-**user**		**Low**-**dose aspirin user**		**d** ^**†**^	**Aspirin non**-**user**		**Low**-**dose aspirin user**		**d** ^**†**^
		**(N = 244,882)**		**(N = 16,183)**			**(N = 15,849)**		**(N = 15,849)**		
		**N**	**(%)**	**N**	**(%)**		**N**	**(%)**	**N**	**(%)**	
Gender	Male	144,933	(59.2)	9,192	(56.8)	0.0483	8,877	(56.0)	8,986	(56.7)	0.0139
	Female	99,949	(40.8)	6,991	(43.2)		6,972	(44.0)	6,863	(43.3)	
Age	40-49	73,569	(30.0)	3,806	(23.5)	0.3481	3,631	(22.9)	3,737	(23.6)	0.0158
	50-59	76,267	(31.1)	5,165	(31.9)	0.0356	4,690	(29.6)	5,067	(32.0)	0.0516
	60-69	57,709	(23.6)	4,433	(27.4)	0.1971	4,489	(28.3	4,342	(27.4)	0.0207
	70-99	37,337	(15.2)	2,779	(17.2)	0.1385	3,039	(19.2)	2,703	(17.1)	0.0551
Insurance type	Health insurance	225,785	(92.2)	14,896	(92.0)	0.0212	14,032	(88.5)	14,615	(92.2)	0.1250
	Medicaid	15,399	(6.3)	947	(5.9)	0.0779	1,334	(8.4)	920	(5.8)	0.1018
	Switching	3,698	(1.5)	340	(2.1)	0.3086	483	(3.0)	314	(2.0)	0.0681
Type of diabetes	Type 1 only	10,505	(4.3)	910	(5.6)	0.0615	1,011	(6.4)	809	(5.1)	0.0548
	Type 2 and others	234,377	(95.7)	15,273	(94.4)		14,838	(93.6)	15,040	(94.9)	
Antidiabetic medication	OHA	206,194	(84.2)	14,315	(88.5)	0.1241	13,078	(82.5)	14,101	(89.0)	0.1854
	OHA + insulin	38,688	(15.8)	1,868	(11.5)		2,771	(17.5)	1,748	(11.0)	
Diagnosis of essential hypertension	Yes	56,688	(23.1)	7,912	(48.9)	0.5566	8,379	(52.9)	7,624	(48.1)	0.0954
Diagnosis of dyslipidemia	Yes	27,592	(11.3)	4,253	(26.3)	0.3918	4,601	(29.0)	4,072	(25.7)	0.0749
Medication use	Statins	28,601	(11.7)	4,283	(26.5)	0.3832	4,291	(27.1)	4,076	(25.7)	0.0308
	ACEI	11,878	(4.9)	2,266	(13.8)	0.3102	2,194	(13.8)	2,028	(12.8)	0.0308
	ARB	24,597	(10.0)	4,321	(26.7)	0.4404	4,301	(27.1)	4,063	(25.6)	0.0341
	CCB	47,268	(19.3)	6,311	(39.0)	0.4439	6,552	(41.3	6,099	(38.5)	0.0584
	Beta blockers	26,413	(10.8)	2,696	(16.7)	0.1713	3,291	(20.8)	2,624	(16.6)	0.1082
	Thiazide diuretics	30,824	(12.6)	4,481	(27.7)	0.3834	4,811	(30.4)	4,264	(26.9)	0.0764
	NSAID	145,733	(59.5)	10,155	(62.8)	0.0665	10,416	(65.7)	9,939	(62.7)	0.0628

d: standardized difference.

ACEI: angiotensin converting enzyme inhibitor; ARB: angiotensin receptor blocker; CCB: calcium channel blocker; NSAID: non-steroidal anti-inflammatory drugs; OHA: oral hypoglycemic agents SD: standard deviation.

*Baseline characteristics for study subjects were identified within one year before index date.

^†^Standardized difference (d) of greater than 0.1 represents meaningful imbalance between study groups.

Table 2 shows the incidence rates and risk of ischemic stroke associated with the use of low-dose aspirin in the propensity score matched cohort. Of the low-dose aspirin users, 340 (2.15%) patients had an ischemic stroke compared with 158 (1.00%) in the matched aspirin non-users. The overall ischemic stroke rate was 5.4 per 1,000 person-years for aspirin users and 3.2 per 1,000 person-years for aspirin non-users. Using a Cox proportional hazards model, compared to aspirin non-users, the adjusted hazard ratio of aspirin users for hospitalization for ischemic stroke was 1.73 (95% confidence interval; 1.41-2.12).

**Table 2:** **Incidence rates and risk of ischemic stroke associated with use of low**-**dose aspirin among patients with diabetes**

		**Aspirin non-user**			**Low-dose aspirin user**				
		**Event (N)**	**Person-years**	**Crude incidence rate (/1,000 person-years)**	**Event (N)**	**Person-years**	**Crude incidence rate (/1,000person-years)**	**Crude hazard ratio (95%CI)**	**Adjusted hazard ratio** ^*^ **(95% CI)**
Whole matched cohort		158	48,725	3.2	340	44,592	5.4	1.64 (1.32-1.98)	1.73 (1.41-2.12)
Gender	Male	81	27,565	2.9	137	25,224	5.4	1.86 (1.56-2.23)	1.93 (1.46-2.55)
	Female	77	21,160	3.6	103	19,368	5.3	1.45 (1.08-1.95)	1.52 (1.13-2.15)
Age	40-69	85	39,758	2.1	136	37,060	3.7	1.69 (1.28-2.22)	1.81 (1.37-2.38)
	70-99	73	8,967	8.1	104	7,867	11.6	1.65 (1.23-2.23)	1.72 (1.27-2.33)
Type of diabetes	Type 1 only	14	3,078	4.5	19	2,287	8.3	1.74 (0.87-3.47)	1.82 (0.90-3.68)
	Type 2 and others	144	45,647	3.2	221	42,305	5.2	1.61 (1.31-1.99)	1.73 (1.40-2.14)
Diabetes with essential hypertension	Yes	97	24,791	3.9	135	21,306	6.3	1.59 (1.23-2.07)	1.69 (1.30-2.20)
	No	61	23,934	2.5	105	23,286	4.5	1.70 (1.24-2.33)	1.81 (1.32-2.51)
Diabetes with dyslipidemia	Yes	39	13,501	2.9	48	11,255	4.3	1.44 (0.94-2.14)	1.68 (1.09-2.57)
	No	119	35,224	3.4	192	33,337	5.8	1.65 (1.31-2.08)	1.75 (1.39-2.21)

CI: confidence interval.

^*^Adjusted Hazard Ratio calculated using Cox proportional hazard model adjusting for insurance type, anti-diabetic medications at baseline, use of statins, angiotensin converting enzyme inhibitors, angiotensin receptor blockers, beta blockers, calcium channel blockers and thiazide diuretics during follow-up.

We did not find any changes in risk related to gender, age group, and presence of hypertension, dyslipidemia from the results of subgroup analyses. Although point estimate for the risk was relatively low in female patients, the use of low-dose aspirin showed an increased risk of ischemic stroke in male patients (aHR 1.93, 95% CI; 1.46-2.55) or in female patients (aHR 1.52, 95% CI; 1.13-2.15). In the type of diabetes, type 1 only group was not statistically significant (aHR 1.82, 95% CI; 0.90-3.68). Use of aspirin increased the risk of hospitalization for an ischemic stroke regardless of the presence of comorbidities. The HRs for the patients with hypertension, without hypertension, with dyslipidemia, or without dyslipidemia were 1.69 (95% CI; 1.30-2.20), 1.81 (95% CI; 1.32-2.51), 1.68 (95% CI; 1.09-2.57), or 1.75 (95% CI; 1.39-2.21), respectively.

In a sensitivity analysis of study subjects with more than 1 year follow-up periods, we found slightly higher aHR (1.97, 95% CI; 1.51-2.62). The aHR for ischemic stroke was 3.25 (95% CI; 2.28-4.63) among males and 1.72 (95% CI; 1.16-2.57) among females. The association between the use of aspirin and the risk of hospitalization due to ischemic stroke maintained irrespective of gender, age, or comorbidities (Table 3).

**Table 3:** **Sensitivity analysis for incidence rates and risk of ischemic stroke associated with use of low**-**dose aspirin among patients with diabetes who completed more than 1 year follow**-**up periods after index date**

		**Aspirin non**-**user**			**Low**-**dose aspirin user**				
		**Event (N)**	**Person-years**	**Crude incidence rate (/1,000 person-years)**	**Event (N)**	**Person-years**	**Crude incidence rate (/1,000 person-years)**	**Crude Hazard Ratio (95% CI)**	**Adjusted Hazard Ratio** ^*^ **(95% CI)**
Whole matched cohort		84	48,553	1.7	139	44,527	3.1	1.85 (1.41-2.44)	1.97 (1.51-2.62)
Gender	Male	42	27,476	1.5	78	25,182	3.1	3.01 (2.12-4.28)	3.25 (2.28-4.63)
	Female	42	21,077	2.0	61	19,345	3.2	1.65 (1.65-2.45)	1.72 (1.16-2.57)
Age	40-69	48	39,633	1.2	85	37,019	2.3	1.98 (1.98-2.62)	2.12 (1.48-3.03)
	70-99	36	8,921	4.0	54	7,508	7.2	1.79 (1.79-2.74)	1.89 (1.23-2.91)
Type of diabetes	Type 1 only	8	3,064	2.6	8	2,282	3.5	1.32 (0.50-3.52)	1.38 (0.50-3.77)
	Type 2 and others	76	45,489	1.7	131	42,245	3.1	1.91 (1.44-2.54)	2.05 (1.54-2.74)
Diabetes with essential hypertension	Yes	48	24,669	1.9	82	21,272	3.9	2.03 (1.42-2.91)	2.14 (1.50-3.07)
	No	36	23,885	1.5	57	23,225	2.5	1.67 (1.09-2.54)	1.78 (1.16-2.73)
Diabetes with dyslipidemia	Yes	24	13,444	1.8	27	11,240	2.4	1.34 (0.78-2.34)	1.60 (0.92-2.82)
	No	60	35,109	1.7	112	33,287	3.4	2.04 (1.49-2.80)	2.13 (1.55-2.93)

CI: confidence interval.

^*^Adjusted Hazard Ratio calculated using Cox proportional hazard model adjusting for insurance type, anti-diabetic medications at baseline, use of statins, angiotensin converting enzyme inhibitors, angiotensin receptor blockers, beta blockers, calcium channel blockers and thiazide diuretics during follow-up.

This retrospective cohort study showed that there was increased the risk of low-dose aspirin use for hospitalization due to ischemic stroke among incident diabetic patients without a prior history of cardiovascular diseases in a real-world setting. Moreover, we did not observe any differences in risk related to gender, age group, or presence of hypertension, or dyslipidemia in subgroup analyses and in a sensitivity analysis of study subjects with more than 1 year follow-up periods. These results suggest that the low-dose aspirin use for the primary prevention of ischemic stroke is unnecessary for patients with diabetes.

Contrary to our initial hypothesis, we found that use of low-dose aspirin increased the risk of hospitalization for ischemic stroke. Diabetes itself might be contributed to reduction of the effectiveness of aspirin due to accelerate platelet turnover [15-18]. Recently, De Berardis et al. reported that the use of aspirin was not associated with greater risk of major bleeding among patients with diabetes [19]. In this respect, the effect of aspirin in patients with diabetes was insufficient not only for preventing cardiovascular events but also for increasing the risk of bleeding. A higher dosing strategy has been proposed by some to overcome these changes in patients with diabetes based on the significant results of the Early Treatment of Diabetic Retinopathy Study trial (650 mg/day) [20]. Henry et al. suggested that repeated low-dose aspirin use daily instead of higher dosing strategy to improve clinical efficacy on patients with aspirin resistance [21]. We did not determine the effect of aspirin dosing due to restricting exposure to low-dose aspirin as current guidelines. Additional research is required to assess the effect of dose escalation for resolving aspirin resistance and the occurrence of adverse events in diabetic patients.

Our findings on the effects of ischemic stroke in diabetic patients correspond to the results of previous observational studies [22,23]. Leung et al. [22] conducted a longitudinal observational study to examine the benefit and harm of low-dose aspirin (75-325 mg/day) in Chinese type 2 diabetic patients. In a total of 5 731 patients, the use of aspirin was associated with increased risk of cardiovascular diseases and mortality in primary prevention (HR 2.07, 95% CI; 1.66-2.59) during the five-year follow-up. In addition, the study showed that the risk of gastrointestinal bleeding in aspirin users was rather high. A Swedish record linkage study [23] was performed to evaluate the effect of aspirin on mortality and serious bleeding in diabetic patients with and without cardiovascular diseases. During the maximum 18 months of follow-up, aspirin significantly increased mortality in the diabetic patients without cardiovascular disease. However, aspirin tended to decrease mortality among elderly diabetic patients with cardiovascular disease. Recently, Sacco et al. [24] performed prospective multicenter study, the use of low-dose aspirin was ineffective in primary prevention of major adverse cardiovascular events for patients with nephropathy (HR: 1.11 95% CI 0.91-1.35). On the other hand, the Fremantle Diabetes Study (FDS) [25] showed that regular aspirin use was independently associated with reduced cardiovascular disease mortality (HR 0.30, 95% CI; 0.09-0.95) and all-cause mortality (HR 0.53, 95% CI; 0.28-0.98) among the 651 low-dose aspirin users (7.7%) without prior cardiovascular disease. There was a possibility of selection bias in recruiting the FDS study participants: 1,426 were recruited to the cohort voluntarily among the postal code-defined urban community-dwellers with diabetes (2,258 patients). Previous studies provided explanations for the failure (non-responsiveness, resistance) of aspirin in the primary prevention of diabetic patients [15-18,22,23].

This study had several strengths. The study reflects the real world situation among a nationwide representative population, which covered all diabetic patients using the national health insurance claims database in Korea. For reducing bias due to confounding by indication, we restricted study subjects to those who were without any diagnosis or medications history of cardiovascular disease [26], and we applied advanced statistical methods like propensity score [12]. We identified potential confounding factors including pre-existing medical conditions and use of medication in the year prior to the index date. We included the insulin or oral hypoglycemic use as a proxy indicator for the severity of diabetes, and the presence of COPD as a proxy for the smoking. We also comprehensively considered widely used medications in relatively recent years such as statin and anti-hypertensives. The HIRA database is a valuable data source for epidemiologic research; the overall positive predictive value of the diagnosis was about 70% [27]. Diagnosis of severe condition such as ischemic stroke was reported to be higher accuracy for claims compared with those of other mild conditions [28]. In particular, diagnosis of diabetes (ICD-10 codes E10–14) in HIRA database was reported to have a positive predictive value of 72.3% in outpatients and 87.2% in inpatients compared to the clinical information obtained by using hospital medical records [29]. We considered both diagnosis and prescription to guarantee a more accurate definition of diabetic patients.

However, these results must be interpreted in light of some potential limitations. One is that despite applying advanced statistical methods like propensity score matching to reduce the effect of confounding, it could not completely rule out bias such as confounding by indications and unmeasured confounders. We could not consider variables that were not included in the claims database such as obesity, smoking, alcohol consumption, or use of over-the-counter drugs. Aspirin is available as an over-the-counter drug and prescription drug in South Korea. We assumed that most patients with diabetes received low-dose aspirin with a prescription during regular clinical visits for managing their diabetes because low-dose aspirin applied to health insurance coverage is discounted compared to over-the-counter self-medication. Accordingly, very few patients bought aspirin at the drug store as over-the-counter drug. We identified very low use of aspirin among the study subjects. Park IB et al. [30] also reported that significant underuse of aspirin therapy among the newly diagnosed diabetes in Korea previously.

Current guidelines for managing diabetes mellitus, aspirin is no longer recommended for CVD prevention at low CVD risk considering the balance of benefit and risk [31]. Based on our results, low-dose aspirin might be not effective with early-stage of diabetes. However, we did not follow-up those patients for a long time, therefore, this results should be interpreted with caution. Up to now, many retrospective studies have been published. On current evidence-conflicting stage, clinicians should evaluate the balance of benefit and risk of low-dose aspirin considering the prevalence period with diabetes. To confirm this issue, randomized controlled trial should be performed for generating strong evidence. Further studies of large-scale intervention trials [32,33] currently in progress will provide more confirmation of the role of low-dose aspirin for the primary prevention of cardiovascular diseases in patients with diabetes.

## Conclusions

In summary, the use of low-dose aspirin was associated with the development of ischemic stroke in diabetic patients without cardiovascular diseases after restricting study subjects and propensity score matching. These results suggested that low-dose aspirin use for the primary prevention of ischemic stroke should be reconsidered in diabetic patients.

## Abbreviations

ACEI: Angiotensin converting enzyme inhibitors
ARB: Angiotensin receptor blockers
BB: Beta blockers
CCB: Calcium channel blockers
CI: Confidence interval
HIRA: Health Insurance Review and Assessment Service
HR: Hazard ratio
ICD-10: International Classification of Disease and Related Health Problems, 10th Revision
NHI: National Health Insurance
NSAIDs: Non-steroidal anti-inflammatory drugs
PPP: Primary Prevention Project
